# Immune and Inflammatory Processes in Obesity, Insulin Resistance, Diabetes, and Related Cardiometabolic Complications

**DOI:** 10.1155/2014/579560

**Published:** 2014-09-21

**Authors:** Joseph Fomusi Ndisang, Sharad Rastogi, Alfredo Vannacci

**Affiliations:** ^1^Department of Physiology, University of Saskatchewan College of Medicine, 107 Wiggins Road, Saskatoon, SK, Canada S7N 5E5; ^2^Division of Cardiovascular Medicine, Department of Medicine, Henry Ford Heart & Vascular Institute, 2799 West Grand Boulevard, Detroit, MI 48202-2689, USA; ^3^Department of Pharmacology, Center for Integrative Medicine, Center for Molecular Medicine (CIMMBA), University of Florence, Viale Pieraccini 6, 50139 Florence, Italy

The high prevalence of obesity and diabetes in developed and developing nations poses a great health challenge [1, 2]. Obesity is one of the major causes of insulin resistance and type-2 diabetes [3]. Type-1 diabetes is primarily due to the autoimmune-mediated destruction of pancreatic beta-cell leading to insulin deficiency [4, 5]. This is generally accompanied by alterations in lipid metabolism, enhanced hyperglycemia-mediated inflammation and oxidative stress, endothelial cell dysfunction, and apoptosis. Similarly, type-2 diabetes is characterized by elevated inflammation, glucotoxicity, lipotoxicity, and apoptosis that leads to the progressive loss of beta cells and ultimately to insulin insufficiency at later stages of the disease [4, 5]. Thus, in diabetes, inflammation could be triggered by hyperglycemia and/or immune response. However, elevated inflammatory events not only may affect insulin production in type-2 and type-1 diabetes but also may affect insulin response in target tissues causing insulin resistance [3]. Although insulin resistance has traditionally been associated with type-2 diabetes, mounting evidence indicates that the incidence of insulin resistance in type-1 diabetes is increasing. Therefore, novel mechanistic approaches deciphering the role of inflammation in insulin resistance in type-1 and type-2 diabetes are needed. Many pathophysiological agents are implicated in insulin resistance. Although the exact nature of these factors is not completely understood, a high consensus of opinion suggests that inflammation, oxidative stress, and genetic, habitual, environmental, and epigenetic factors are implicated.

There has been significant advancement in elucidating the mechanisms implicated in insulin resistance, overt diabetes, and related cardiometabolic diseases [1–9]. However, novel mechanistic studies deciphering the role of inflammation in these chronic diseases are needed. Similarly, novel studies addressing the effect of inflammation on genetic and epigenetic factors that lead to insulin resistance, overt diabetes, and related cardiometabolic complications are needed. Therefore, this special issue highlights research and review papers that address a wide spectrum of inflammation-related mechanisms associated with insulin resistance, type-1 diabetes, type-2 diabetes, and related cardiometabolic complications. Accordingly, in an article featuring in this special issue, L. Zhang and coworkers investigated the pathophysiological role of tribbles homolog-3 (TRB3) in diabetic nephropathy, a common complication of diabetes. The authors reported that TRB3 may trigger renal fibrosis by regulating transforming growth factor *β*1 (TGF-*β*1) and collagen type-IV through a signaling pathway involving extracellular signal-regulated kinase and mitogen-activated protein kinase. TGF-*β* is a glycoprotein and cytokine with diverse roles in many cellular events including reproduction [10]. On the other hand, gamma interferon (IFN-*γ*), another cytokine that is traditionally known for its role in innate and adaptive immunity, is increasingly reported to play a role in reproduction [11]. In this special issue, D. L. G. Fagundes et al. showed that IFN-*γ* and TGF-*β* modulate the phagocytic activity in the colostrum, maternal blood, and cord blood of pregnant diabetic women. There is an interesting reciprocal interaction between TGF-*β* and macrophage migration inhibitory factor (MIF) [12]. MIF is a proinflammatory cytokine that promotes immune cell recruitment following injury and polymorphism of MIF which has been associated with several diseases [13, 14]. In a related study by E. Valdés-Alvarado et al., the association of MIF gene polymorphism, a complication that is commonly associated with obesity, diabetes, and hypertension, and susceptibility to acute coronary syndrome was reported in a research article of this special issue. Furthermore, in a clinical study by N. A. Sinicato and coworkers, the role of cytokines such as tumor necrosis factor alpha, interleukin- (IL-) 6, and interleukin- (IL-) 10 in systemic lupus erythematosus, an autoimmune disease that is associated with a variety of different cardiovascular complications including atherosclerosis, was reported. Many cytokines are known to potentiate inflammatory cascades by modulating macrophage polarization [15]. The role of the different macrophage M1 and M2 phenotypes in obesity is becoming increasingly clear [3, 15, 16]. In a related article featuring in this special issue, K. Fjeldborg et al. have shed more light on the preponderance of macrophage M2 phenotype that was associated with a parallel reduction of the macrophage M1 phenotype in obese subjects. Macrophage-induced inflammation remains an important feature in insulin resistance and type-2 diabetes; thus, as an alternative strategy, A. L. Guadarrama-López et al. underscored the beneficial effects of polyunsaturated fatty acids and vitamin D in diabetes and related complications in a review article contained in this issue.

Diabetic retinopathy is another complication of diabetes and is amongst the leading causes of vision impairment [17] and a significant number of patients with diabetic retinopathy are also known to be affected by diabetic macular edema [18]. A common denominator between diabetic retinopathy and diabetic macular edema is the elevated levels of role of vascular endothelial growth factor (VEGF) [19, 20]. In this special issue, a review article by F. R. Stefanini and coworkers is featured highlighting the role of intravitreal injection of anti-VEGF as a therapeutic strategy against diabetic macular edema.

Collectively the articles featuring in this special issue constitute a cocktail of original research and reviews that would stimulate further research in this area given the increasing incidence of diabetes, obesity, hypertension, and the burden these chronic conditions pose to health care systems.

*Joseph Fomusi Ndisang* *Joseph Fomusi Ndisang* *Sharad Rastogi* *Sharad Rastogi* *Alfredo Vannacci* *Alfredo Vannacci*
